# Dengue-induced pancarditis manifestation of young female with systemic lupus erythematosus: case report

**DOI:** 10.1093/ehjcr/ytag211

**Published:** 2026-03-18

**Authors:** Mochamad Rizky Hendiperdana, Muhammad Adityansah, Astrid Putri, Nugroho Sigit

**Affiliations:** Division of Cardiovascular Medicine, Pandan Arang General Hospital, Kantil Road No.14, Boyolali, Central Java 57316, Indonesia; Division of Cardiovascular Medicine, Pandan Arang General Hospital, Kantil Road No.14, Boyolali, Central Java 57316, Indonesia; Division of Cardiovascular Medicine, Pandan Arang General Hospital, Kantil Road No.14, Boyolali, Central Java 57316, Indonesia; Department of Radiology, Pandan Arang General Hospital, Kantil Road No.14, Boyolali, Central Java 57316, Indonesia

**Keywords:** Lupus, Dengue, Pancarditis, Myocarditis, Heart failure, Case report

## Abstract

**Background:**

Lupus activity is associated with cardiac manifestation with varied clinical spectrum from asymptomatic disease-to-fulminant heart failure (HF) and cardiogenic shock. Pancardiac inflammation manifestation in systemic lupus erythematosus (SLE) patient with acute HF manifestation is relatively rare condition. Although, the most common cardiac involvement of SLE is pericarditis. However, cardiac involvement of SLE can affect all layers of the heart: endocardium as valvulitis, myocardium, and pericardium. Overt lupus myocarditis was a rare occurrence (5%–10%), but has a fatal outcome, and this was associated with worse prognostic. While, valvulitis manifestation of SLE is also rare compared to pericarditis, it accounts for less than 20% of SLE.

**Case summary:**

A 39-year-old female presented with acute HF symptom with vasculitis sign phenomena and renal insufficiency. Laboratory result showed leukocytopenia and thrombocytopenia. Echocardiography finding marked for valvulitis, myocarditis, and pericarditis involvement. Rheumatology work-up was significant for SLE with high disease activity. Other serology work-up showed positive dengue virus IgM. The patient was diagnosed with dengue-induced pancarditis with lupus nephritis. The patient was administered corticosteroid, HF medication, and cyclosporin. After 3 months follow-up, we found significant improvement in clinical and echocardiography functional parameter.

**Discussion:**

Systemic lupus erythematosus is a chronic systemic autoimmune disease, and the proposed mechanism of lupus flare in our case is related to dengue-triggered immunological activation. There are enormous reported cases of complete myocardial function recovery after several months of steroid therapy in SLE with cardiac manifestation. The improvement of functional imaging parameters reflected a favourable clinical response to immunosuppressive therapy.

Learning points

- This case highlighted that lupus flare could be induced by dengue virus infection and resulting in cardiovascular manifestation.
- Lupus pancarditis, though a rare condition, has fatal consequence such as heart failure manifestation with high mortality if untreated.
- Early detection and appropriate management of immunosuppressive for primary insult of SLE become the key management approach in this case for instantaneous clinical improvement.

## Introduction

Lupus activity is associated with cardiac manifestation with varied clinical spectrum from asymptomatic disease-to-fulminant heart failure (HF).^1–3^ Pancardiac inflammation manifestation in systemic lupus erythematosus (SLE) patient with acute HF manifestation is relatively rare condition. Although the most common cardiac involvement of SLE is pericarditis, cardiac involvement of SLE can affect all layers of heart: endocardium as valvulitis, myocardium, and pericardium.^4^ Overt lupus myocarditis (LM) was a rare occurrence (5%–10%), but has a fatal outcome, and this was associated with worse prognostic. While valvulitis manifestation of SLE is also rare compared to pericarditis, it accounts for less than 20% of SLE.^1–6^

## Summary figure

Visual summary of pathophysiological process.

**Figure:**
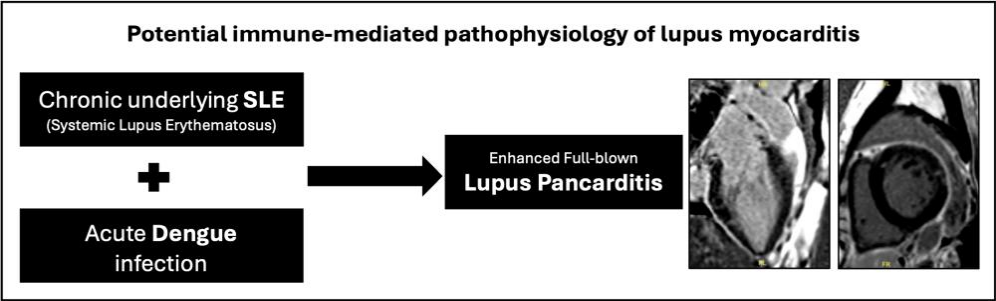


## Case summary

A 39-year-old female presented to emergency ward with progressive shortness of breath that worsened 3 days prior to present admission. The patient complained peripheral leg oedema, abdominal pain that accompanied by constitutional symptom of febrile, joint pain, hair loss, progressive weight loss of 7 kg in a year, amenorrhoea for a year, and scattered painless redness on palmar and plantar fingers several weeks prior to recent admission. The patient has no remarkable medical history except for intermittent joint pain. The patient has no history of hypertension and no signs of suggestive SLE.

**Figure 1:**
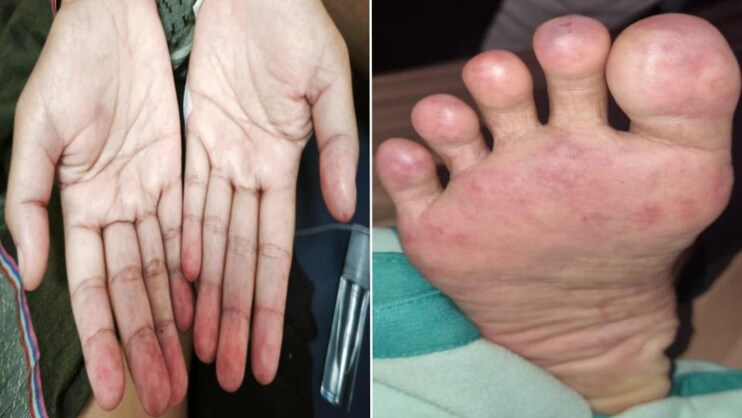
Vasculitis erythematous sign on palmar (left) and plantar (right) fingers area.

**Figure 2:**
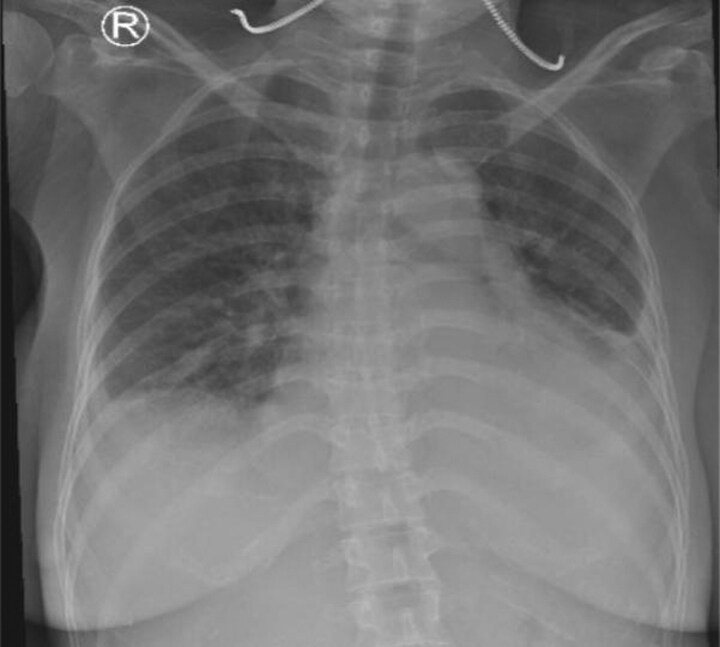
Chest X-ray demonstrated cardiomegaly, bilateral pleural effusion, and pulmonary vascular cephalization.

On admission, vital signs were as follows: blood pressure 160/100 mmHg, heart rate 86 b.p.m., peripheral oxygen saturation 98%, temperature of 38.5°C, and respiratory rate 22 x/minute. Physical examinations were notable for lung rales and early diastolic murmur at aortic valve area on auscultation with apical displacement on palpation and bilateral pretibial oedema with vasculitis erythematous sign on palmar and plantar fingers (*Figure 1*). Other findings were unremarkable. Electrocardiography showed sinus rhythm with non-specific R wave progression sign. Chest X-ray demonstrated cardiomegaly, bilateral pleural effusion, and pulmonary vascular cephalization (*Figure 2*).

Laboratory test remarkable findings were as follows: Hb 10.8 g/dL, leukocyte 3260/μL, thrombocyte 65 000/μL, erythrocyte sedimentation rate (ESR) 21 mm/h, serum creatinine 2.3 mg/dL, serum albumin 3.4 mg/dL, D-dimer 3032 ng/mL, euthyroid, non-reactive HBsAg, and positive anti-dengue IgM. Urinalysis finding was significant for haematuria and proteinuria. Echocardiography revealed dilatation of all heart chamber with reduced left ventricular ejection fraction (LVEF) of 25% and global hypokinetic and reduced TAPSE to 14 mm. Moderate aortic and tricuspid valve regurgitation were also observed with pericardial effusion also noted (see Supplementary material online, *Video S1*).

Due to high suspicious of cardiac involvement of autoimmune disease, the patient’s rheumatological work-up was performed. The patient ANA test was positive, and ANA profile showed high likelihood for SLE diagnosis (*Table 1*). Hence, the patient was diagnosed as dengue-induced SLE pancarditis with acute HF presentation and lupus nephritis (LN). The lupus diagnosis was based on EULAR/ACR criteria with total score of 22, whereas value > 10 is classified as SLE.^7^ Initial Mex-SLEDAI score was 27 which signify for severe disease activity.

**Table 1:** Anti-nuclear antibody profile

Anti-nuclear antibody profile	Class
Anti-RNP/SM	0 (negative)
Anti-Sm	(+) (borderline)
Anti-SS-A native (SSA)	+++ (strongly positive)
Anti-R0-52 recombinant	+++ (strongly positive)
Anti-SS-B (SSB)	+++ (strongly positive)
Anti-Scl-70 (Scl)	0 (negative)
Anti-PM Scl-100 (PM)	0 (negative)
Anti-Jo-1 (Jo)	0 (negative)
Anti-centromere B (CB)	0 (negative)
Anti-PCNA	0 (negative)
Anti-dsDNA (DNA)	0 (negative)
Anti-nucleosomes (NUC)	+++ (strongly positive)
Anti-histones (HI)	+++ (strongly positive)
Anti-ribosomal protein (RIB)	0 (negative)
Anti-AMA-M2 (M2)	+ (positive)
Anti-DFS70	0 (negative)
Control (ko)	+++ (strongly positive)

Upon to this finding, patient was administered intravenous methylprednisolone 20 mg t.i.d., intravenous loop diuretic furosemide 20 mg/hour, and HF medication: ramipril 5 mg o.d., bisoprolol 2.5 mg o.d., and spironolactone 25 mg o.d. On the 3rd admission day, clinical improvement was observed. Heart failure medications were up-titrated to ramipril 10 mg o.d. and bisoprolol 5 mg o.d. The patient was discharged on the 5th admission day uneventfully. The oral methylprednisolone was continued with regimen 16 mg twice daily.

**Figure 3:**
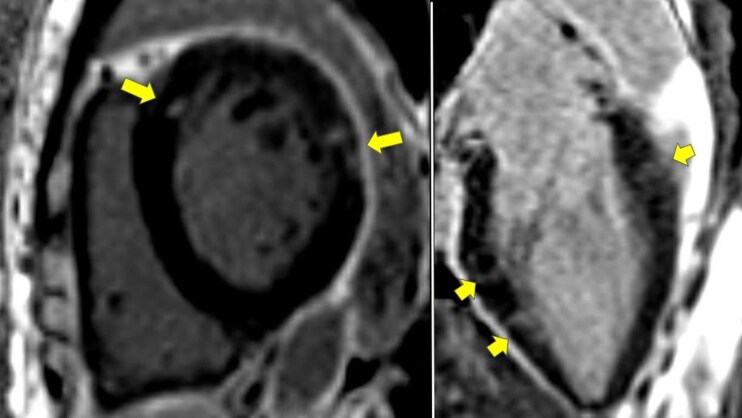
Cardiac magnetic resonance imaging showed mid-wall late gadolinium enhancement at the anteroseptal and anterolateral segments and subepicardial late gadolinium enhancement at the basal lateral segment which signify for non-ischaemic LGE (yellow arrow).

One week after discharge, cardiac magnetic resonance (CMR) imaging was performed, and we found reduced ejection fraction of 30% with global hypokinetic in LV segment with minimal myocardial oedema. Midwall late gadolinium enhancement (LGE) was also observed at the anteroseptal and anterolateral segments and subepicardial LGE at the basal lateral segment (*Figure 3*). This finding suggested non-ischaemic LGE and strengthened the myocarditis finding. The patient was administered cyclosporine 50 mg p.o. twice daily for additional immunosuppressive therapy. We observed improvement in renal function during follow-up course up to 2 months post discharge (*Table 2*).

**Table 2.** Follow-up laboratory parameters

	19 May (admission)	22 May (discharge)	17 June	30 June	24 July
Haematology					
Haemoglobin (g/dL)	10.8	10.5	10.4	10.4	12.1
Leukocyte (cells/µL)	3260	3300	2900	5570	4130
Thrombocyte (cells/µL)	65 000	59 000	106 000	102 000	149 000
Blood chemistry					
Serum creatinine (mg/dL)	2.3	3.1	2.1	1.8	1.6
Estimated GFR (mL/minute)	25.09	16.70	21.54	33.29	38.14
Blood urea nitrogen (mg/dL)	84	140	78	66	67
SGOT (U/L)	19				
SGPT (U/L)	5				
Urinalysis					
Urine protein	+ 3		+3	+3	
Blood	+ 3		+1	+1	
Nitrite urine	Negative		Negative	Negative	
Leukocyte esterases	Negative		Negative	Negative	
Erythrocyte sediment	+2			+1	
Epithelial sediment	+1			+1	

GFR, glomerular filtration rate; SGOT, serum glutamic oxaloacetic transaminase; SGPT, serum glutamic pyruvic transaminase.

In three months post-discharge evaluation, we observed remarkable clinical and functional improvement. There was no re-hospitalization period from HF during the follow-up time. Echocardiographic parameter improvement was also observed with LVEF of 60% and TAPSE 20 mm; no residual pericardial effusion was noted (see Supplementary material online, *Video S2*). Mex-SLEDAI score decreased to 4, demonstrating excellent treatment response with complete cardiac recovery and substantial disease activity reduction. Heart failure and immunosuppressive medication (methylprednisolone and cyclosporin) were continued.

## Discussion

Systemic lupus erythematosus is a chronic systemic autoimmune disease, and the proposed mechanism of lupus flare in our case is related to dengue-triggered immunological activation.^1,2^ The recent admission of positive antibody for dengue is hypothetically induced hyperactivity of inflammation and causes pancarditis manifestation. Previous reports have reported the occurrence of dengue-induced SLE flare. Rajadhyaksha and Mehra^8^ described SLE with LN manifestation in dengue virus infection. It is important to consider SLE cardiac manifestation especially in young woman patients who developed acute HF that accompanied with vasculitis stigmata and nephritis.^6^

Lupus myocarditis has a poor prognosis with 40% of patients died or having persistent LV systolic dysfunction although adequately managed. The poor outcome and incomplete recovery were associated with the lower LVEF at initial presentation (LVEF < 35%).^2,3^ Furthermore, the presence of LN involvement, which accounts for 40%–70% of SLE and cutaneous signs of SLE like vasculitis, strengthens the association of lupus activity with our case clinical manifestation.^2,3^ Liu *et al.*^9^ reported that a higher Mex-SLEDAI score is an independent risk factor contributing to development of myocarditis in SLE patients. Our case also showed high Mex-SLEDAI score at initial presentation, and this is associated with high-risk feature of LM.^1^

Shabbir *et al.*^10^ reported an immediate SLE flares in young postpartum patient that manifest as cardiac tamponade and myocarditis. There were previous reported cases of life-threatening cardiogenic shock manifestation of SLE myocarditis.^6,11^ Furthermore, Durrance *et al.*^12^ also reported an acute HF presentation of myopericarditis with valvular dysfunction in SLE patient. Furthermore, isolated myocarditis manifestation of SLE without other system involvement was also been reported by Hutman-Zahler *et al*.^5^

Interestingly, auto antibodies that found in LM and general SLE patients has no different, except for anti-Ro/SSA antibody that reported to be high as 69% in LM compared to only 40% in general SLE patients and also high activity of anti-Ro/SSA predict the LGE finding from CMR in general SLE patients.^3^ This finding showed that anti-Ro/SSA activity is strongly related with myocardial involvement in SLE,^1,2,5^ as observed in our patient whereas the activity of anti-Ro/SSA antibody is very high. These patterns were also found in previous reports of SLE with cardiac manifestation.^5,11–13^

There were previous reported cases of complete myocardial recovery after several months of steroid therapy in LM patients.^5,6^ The significant and rapid improvement was observed after weeks of appropriate medical management with 60% of complete recovery, while 20% of partial recovery.^2^ We observed complete functional recovery of myocardial function in 3 months after initial immunosuppressive and HF medication in this case.

The clinical spectrum in our case is classified by World Health Organization (WHO) as expanded dengue syndrome (EDS). The EDS is a subset of dengue virus infection beyond the traditional classification with unusual clinical manifestation and multi organ involvement, for instance neurological, gastrointestinal, and renal failure, especially cardiovascular involvement such as myocarditis and pericarditis.^14^ This clinical subset occasionally progressed into serious and life-threatening outcome.^14^ As observed in our case with pancardiac involvement and acute HF. It is hypothesized that dengue cardiac manifestation is caused by direct viral attack and/or immune-mediated mechanism involved.^14^ As in our case context, dengue virus infection induced lupus flare that affect pancardiac inflammation. It is hypothesized that dengue and SLE relied on the same pathogenetic and genetic background.^15^ The EDS is under-recognized clinical spectrum. Hence, the vigilance consideration of EDS potentially addressed the appropriate management strategy for dengue with unusual manifestation.^14^

The association between dengue infection and risk of SLE activation had been described by Chen *et al.* from large population-based study. History of dengue infection is found more significantly associated with SLE (OR 4.55; *P* < 0.001) while was not found in other systemic autoimmune rheumatic disease such as Sjogren’s syndrome, systemic sclerosis, and rheumatoid arthritis. This finding had clinical significance concerning short- and long-term effect of dengue infection history for SLE flare that can affect end-organ target especially cardiac tissue.^15^ However, clinical diagnosis establishment of this direct relationship relies on histopathological data from renal and/or skin rash tissue.

With the lack of histopathology data of renal and skin rash, the clinical question emerged whether the clinical syndrome in our patient constitutes a self-resolved dengue-induced pancarditis with false-positive SLE laboratory test or a dengue-induced SLE flare that involves pancardiac inflammation and HF. This becomes the limitation of this case report. However, the establishment of dengue-induced lupus flare diagnosis in our case relied on several objective finding. Firstly, the skin vasculitis that concomitantly occurred during the acute cardiac decompensation strengthens the possibility of autoimmune-mediated clinical syndrome. Second, the EULAR/ACR and Mex-SLEDAI clinical score assessment were highly associated with lupus activity. Lastly, LN finding in our casestrenghtening the association with lupus activity.

Other limitation in our case report was the absence of dengue genomic and serotype identification due to resource constraint. However, to the best of our knowledge, this is a first case report of pancardiac involvement of dengue-induced SLE that responded well with immunosuppressive and HF therapy as recommended.^2^ This condition is potentially to be under-recognized and resulting in delaying diagnosis.^1,12^ Early detection and appropriate management of immunosuppressive for primary insult of SLE become the key management approach.

## Supplementary Material

ytag211_Supplementary_Data

## Data Availability

The data underlying this article are available in the article and in its online supplementary materials.

## Lead author biography

Mochamad Rizky Hendiperdana, M.D, is cardiologist at Pandan Arang General Hospital, Boyolali, Central Java, Indonesia. He completed his cardiovascular medicine training and education at the Faculty of Medicine University of Indonesia/National Cardiovascular Centre Harapan Kita, Jakarta, Indonesia. He has interest in interventional cardiology, acute cardiovascular care, and heart failure topics.

## Notes

**Consent:** The patient provided informed written consent prior to data publication in compliance with COPE guidelines.
